# Comparison between the 0- and 30-s balloon dilation time in percutaneous transluminal angioplasty for restenosed arteriovenous fistula among hemodialysis patients: a multicenter, prospective, randomized trial (CARP study)

**DOI:** 10.1007/s10157-024-02469-8

**Published:** 2024-02-28

**Authors:** Tomoki Saiki, Kensuke Sasaki, Shigehiro Doi, Akira Takahashi, Yosuke Osaki, Naoki Ishiuchi, Yujiro Maeoka, Toru Kawai, Koichiro Kawaoka, Shunsuke Takahashi, Takuo Nagai, Taisuke Irifuku, Ayumu Nakashima, Takao Masaki

**Affiliations:** 1 Department of Nephrology, Hiroshima University Hospital, 1-2-3 Kasumi, Minami-ku, Hiroshima, 734-8551 Japan; 2 Chuo Naika Clinic, Kure, Japan; 3 Department of Nephrology, Kure Kyosai Hospital, Kure, Japan; 4 Department of Nephrology, National Hospital Organization Kure Medical Center, Kure, Japan; 5 Soju Clinic, Hatsukaichi, Japan; 6 Department of Nephrology, National Hospital Organization Higashihiroshima Medical Center, Higashihiroshima, Japan

**Keywords:** Hemodialysis, Vascular access, Percutaneous transluminal angioplasty, Patency rate, Balloon dilation

## Abstract

**Background:**

This study aims to compare patency rates of the 0- and 30-s (sec) balloon dilation time in hemodialysis (HD) patients with restenosis after percutaneous transluminal angioplasty (PTA).

**Methods:**

The patients who underwent PTA within 6 months for failed arteriovenous fistula at the forearm were randomly assigned the 0-s or 30-s dilation time group. Effect of dilation time on the 3- and 6-month patency rates after PTA was examined.

**Results:**

Fifty patients were enrolled in this study. The 3-month patency rate in the 30-s dilation group was better than that in the 0-s dilation group (*P* = 0.0050), while the 6-month patency rates did not show a significant difference between the two groups (*P* = 0.28). Cox’s proportional hazard model revealed that 30-s of inflation time (hazard ratio 0.027; *P* = 0.0072), diameter of the proximal (hazard ratio 0.32; *P* = 0.031), and dilation pressure (hazard ratio 0.63; *P* = 0.014) were associated with better 3-month patency. Dilation pressure between previous and present PTA did not differ in the 0-s (*P* = 0.15) and 30-s dilation groups (*P* = 0.16). The 6-month patency rate of the present PTA in the 30-s dilation group was higher than that of the previous PTA (*P* = 0.015). The visual analog scale did not differ between the two groups (*P* = 0.51).

**Conclusion:**

The presenting data suggest that 30-s dilation potentially results in a better 3-month patency rate than 0-s dilation in HD patients with restenosis after PTA.

## Introduction

Chronic kidney disease (CKD) is a well-recognized global health concern, affecting roughly 800 million individuals worldwide [1]. As CKD progresses, renal function declines, and many patients eventually develop end-stage kidney disease [2], which requires renal replacement therapy, such as hemodialysis (HD), peritoneal dialysis (PD), or renal transplantation [3, 4]. In Japan, the majority of patients undergo HD as their primary renal replacement therapy, as opposed to peritoneal dialysis [5]. However, unlike PD, HD requires vascular access (VA), which is known to be the most common source of complications in HD patients [6, 7]. Therefore, reducing VA-related problems is crucial in decreasing HD-related complications.

Various types of VA are used to perform HD, including arteriovenous fistula (AVF), arteriovenous graft, superficialized artery, and central vein catheter. Among those, AVF is widely recognized as the best form of VA in terms of both patency rates and mortality [8–10]. In fact, the Japanese Society of Dialysis Therapy recommended the creation of AVF as the first choice whenever possible, which is called “the AVF-first policy” [9]. However, VA dysfunction is mainly caused by stenosis, and the 24-month patency rate of AVF in HD patients is reported to be 54.5% [11]. Currently, percutaneous transluminal angioplasty (PTA) is the first-line therapy for stenosed VA. However, the 12-month patency rate of PTA is also reported to be 61.2% [11], necessitating frequent treatment. Therefore, improving the post-PTA patency rate is required to reduce HD-related complications.

The PTA procedure involves several steps, including insertion of the sheath, traversing guide wire through a narrow part of the VA, delivering the catheter, balloon dilation, holding balloon pressure, and deflation. Among those steps, the methods of balloon inflation and deflation, and the duration of balloon dilation may have an impact on post-PTA patency rates. In fact, previous studies have shown that the patency rate does not differ between 1- and 3-min dilation times [12], and that 30-s (sec) dilation exhibits a better patency rate than 1-min dilation [13]. These findings suggest that shorter dilation times may contribute to an increase in patency rates. However, a standard procedure for dilation time has not been established.

In this study, we performed multicenter, prospective, randomized study to compare the 3- and 6-month patency rate between the 0- and 30-s dilation times. We enrolled 51 patients who had undergone PTA within 6 months for failed AVF at the forearm and allocated them to two groups using an allocation table. We also carried out a multivariate Cox proportional hazard model to identify clinical factors that are independently associated with the 3-month patency rate. We compared the dilation pressure between previous and present PTA and compared the 3- and 6-month patency rates between the 0- and 30-s dilation groups. Lastly, we compared the visual analog scale (VAS) between the two groups. The resulting data provide information on the effect of dilation time on the patency rate in HD patients with post-PTA restenosis within 6 months for failed AVF.

## Materials and methods

### Patient population

This study was conducted from 20 September 2016 to 31 March 2021 at 14 facilities in Japan. All patients who met the indication criteria for PTA were considered for entry, as described in the 2011 update Japanese Society for Dialysis Therapy Guidelines of Vascular Access Construction and Repair for Chronic Hemodialysis [9]: 50% stenosis and clinical abnormalities, such as decreased blood flow, aneurysm formation, elevated venous pressure, abnormal high blood urea nitrogen, unexpected reduction of dialysis efficiency, and an abnormal physical condition. Inclusion criteria for this study consisted of patients who underwent a single-lesion PTA procedure on the forearm for a failed AVF within a 6-month period. Exclusion criteria encompassed patients who were younger than 20 years old, those with thrombosed access, those with suspicion of acute infection of access, those who underwent an initial PTA, those with multiple lesions, those with lesions located above the elbow, those who underwent duplicate PTA procedures during the observation period, and those who were unable to provide written informed consent. Patients with allergies to contrast agents, severe heart failure, and pregnancy were also excluded. Indications for PTA were determined based on the assessment of the attending nephrologists. Across the 14 facilities, a total of 8013 PTA procedures (2029 cases) were performed during the observational period. Among those, 66 cases met the indication criteria, and finally, 51 cases were included in this study. Almost all individuals excluded were those who underwent duplicate PTA procedures or those with multiple lesions. This study was conducted in accordance with the Declaration of Helsinki and was approved by the ethics committee of Hiroshima University (approval number: C-113-1). This study was registered in the University Hospital Medical Information Network (UMIN; 000024331) and written informed consent was obtained from all individual participants included in the study.

### Study design

We designed a multicenter, prospective, randomized study comparing two groups. The study aimed to evaluate the patency rates between 0- and 30-s dilation as there is no previous report on this comparison. The target sample size was set at 40 patients for the first evaluation. The study was originally planned to run for 24 months, but due to the low enrollment rate, we extended the registration period by 19 months and increased the number of institutions from 5 to 12. Patients were randomly assigned to either the 0- or 30-s dilation group, using an allocation table that considered sex and the presence of diabetes mellitus (DM).

The patency period was defined as the duration of time from the present PTA to the next PTA or surgical revision. Baseline characteristics collected including age, gender, DM, access creation date, date of the previous PTA, location of the stenosis, diameter of the stenosis site, its proximal and distal veins, and the length of the stenosis. The diameter and the length of stenosis were measured using angiography. Data on dilation pressure, device used, and degree of residual stenosis after treatment were also collected. The observation period was 6-month, during which the rate of complete dilation, initial success rate, incident of complication, and degree of pain using VAS during the PTA procedure were also examined. Considering that previous prospective studies reported an increasing incidence of access failure occurring after 3 months [12, 13], we set a 6-month observation period to prevent an excessively prolonged duration beyond the critical 3-month mark.

### PTA procedure

A 4- or 5-F vascular sheath was inserted toward the stenosis, and heparin was intravenously injected from the sheath at a dose of 2000U. The following sheaths were used: Vaivt A Sheath (Medikit, Tokyo, Japan), Ultrahigh-flow Sheath (Medikit), Mosquito Sincere Catheter Introducer (Boston Scientific Japan, Tokyo, Japan), Goodtec Sheath Introducer (Goodman, Nagoya, Japan). Next, a guidewire was traversed through the stenotic lesion. The following guidewires were used; Radifocus guidewire GT (Terumo, Tokyo, Japan), Kyousha NT (Boston Scientific Japan), Radifocus Guidewire (Terumo), and Buddy Wire (Piolax, Kanagawa, Japan). PTA was performed using a SHIRANUI EX balloon (Kaneka Medical Products, Osaka, Japan) or OHICHOIII balloon (Kaneka) with a diameter of 4–6 mm, which was the same as the previous PTA. The balloon was pressurized by 1 atm every 2 s and expanded to a maximum of 22 atm. Before concluding the procedure, angiography was performed to confirm whether the stenosis was successfully dilated.

### Statistical analysis

The presence of a normal distribution was evaluated by the Shapiro–Wilk test. Data are presented as mean values ± standard deviation (SD) or median and interquartile range (25th–75th percentiles. Continuous variables between the 0- and 30-s dilation groups were compared using the *t* test or the Mann–Whitney *U* test, while categorical variable were compared using the chi-square test. The patency rates were compared using the Kaplan–Meier methods with the log-rank test. Multivariate Cox proportional hazard model was carried out to identify clinical factors that are independently associated with the patency rate. Dilation pressure and VAS was compared using Wilcoxon signed-rank test and Mann–Whitney* U* test, respectively. All analyses were performed using Statistical Package for the Social Sciences software (ver. 21.0; IBM, Armonk, NY, USA). *P* values < 0.05 was considered statistically significant.

## Results

### Patients

**Fig. 1:**
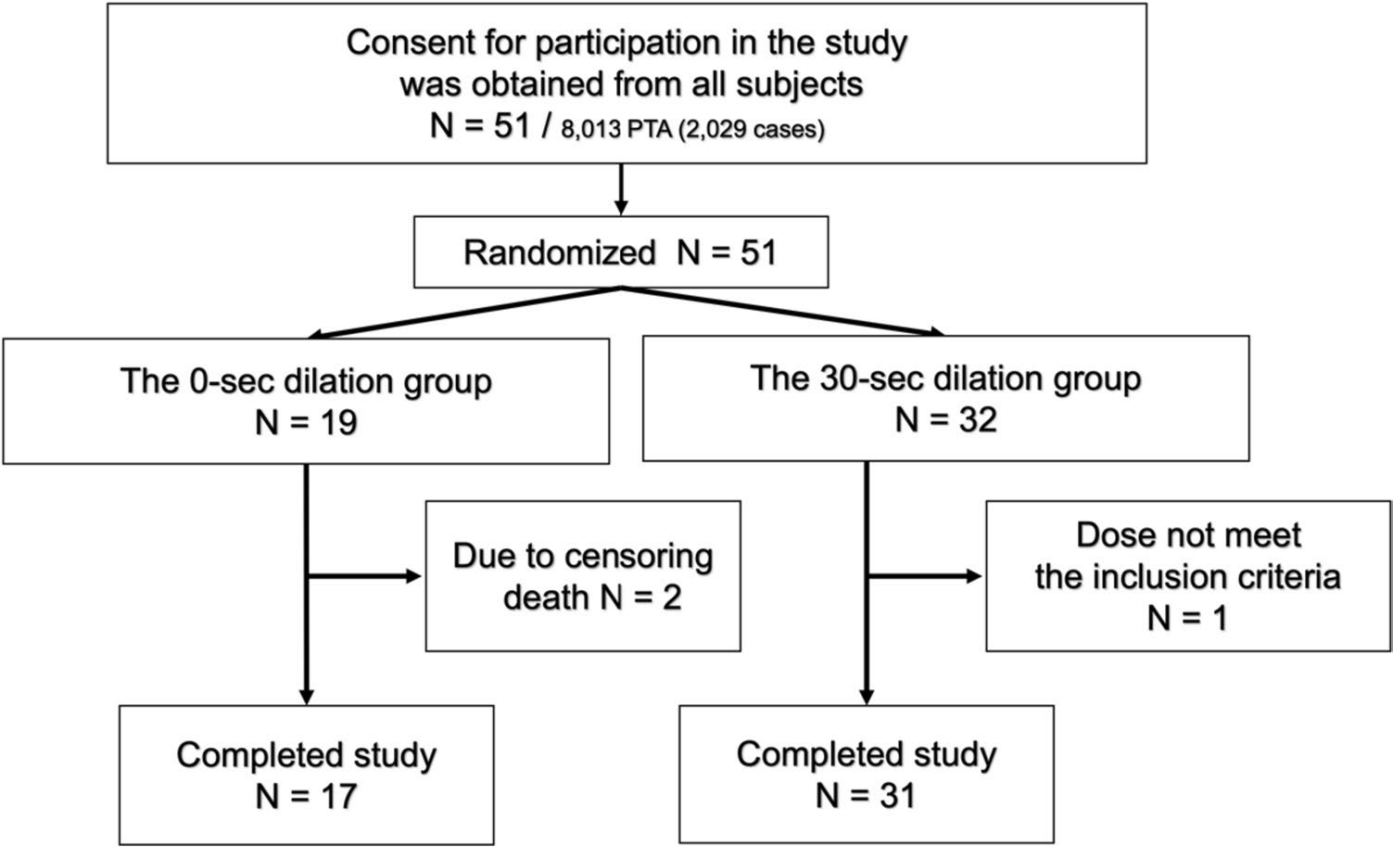
Flow diagram of patient progression through the phases of the present randomized trial. Written informed consent was obtained from a total of 51 patients who participated in this study. After randomization, 19 patients were allocated to the 0-s dilation group and 32 to the 30-s dilation group. All patients successfully underwent PTA. One patient was excluded from the 30-s dilation group due to not undergoing prior PTA. Two patients in the 0-s dilation group were censored due to deaths. Finally, 17 patients in the 0-s group and 31 patients in the 30-s dilation group were completely followed up. PTA: percutaneous transluminal angioplasty

In this study, a total of 51 patients were included, with 19 patients in the 0-s dilation group and 32 patients in the 30-s dilation group, as shown in Fig. 1. One patient in the 30-s dilation group was excluded due to not undergoing prior PTA within 6 months, resulting in a total of 50 patients who underwent successful PTA. No adverse events, such as vascular rupture, were observed during the procedure. However, 2 patients in the 0-s dilation group died during the observation period due to pneumonia and myocardial infarction. The clinical characteristics of all patients are presented in Table 1, which shows that there were no significant differences in age (*P* = 0.54), sex (*P* = 0.13), or the presence of DM (*P* = 0.94). Furthermore, there were no significant differences in diameter of the site of stenosis (*P* = 0.69), stenosis length (*P* = 0.11), and diameter of the proximal (*P* = 0.94) and distal (*P* = 0.88) sides of the stenosis.

**Table 1:** Baseline characteristics of the patients

	All (*n* = 50)	The 0-s inflation (*n* = 19)	The 30-s inflation (*n* = 31)	*P* value
Age (year)	73 [67–82]	77 [63–81]	77 [68–82]	0.54
Gender				
Male (%)	36 (72)	16 (84)	20 (65)	0.13
Diabetes mellitus, Present (%)	24 (48)	9 (47)	15 (48)	0.94
Age of access (day)	764 [480–1528]	705 [414–1425]	997 [590–1585]	0.44
Stenosis diameter				
Site of stenosis (mm)	1.3 [1.0–2.0]	1.2 [0.94–2.3]	1.4 [1.1–2.0]	0.69
Proximal (mm)	5.2 [4.0–6.7]	5.4 [4.0–6.0]	5.0 [4.0–7.1]	0.94
Distal (mm)	4.0 [3.0–5.0]	4.0 [3.1–4.9]	4.0 [2.8–5.7]	0.88
Stenosis length (mm)	14.5 [5.1–28.1]	22.0 [10.0–30.7]	12.0 [5.0–25.0]	0.11
Residual stenosis				
Present (%)	13 (26)	5 (26)	8 (26)	0.90
Degree of stenosis				
Mild (%)	11 (22)	5 (26)	6 (19)	0.56
Moderate (%)	10 (20)	6 (31)	4 (12)	0.11
High grade (%)	29 (58)	8 (42)	21 (67)	0.075
Technical variable				
Balloon pressure (atm)	20 [18–22]	20 [18–22]	20 [16–22]	0.25

Continuous data are summarized as the median [interquartile range] and categorical data as an absolute value (percentage)

### Comparison of patency rates between 0- and 30-s groups

**Fig. 2:**
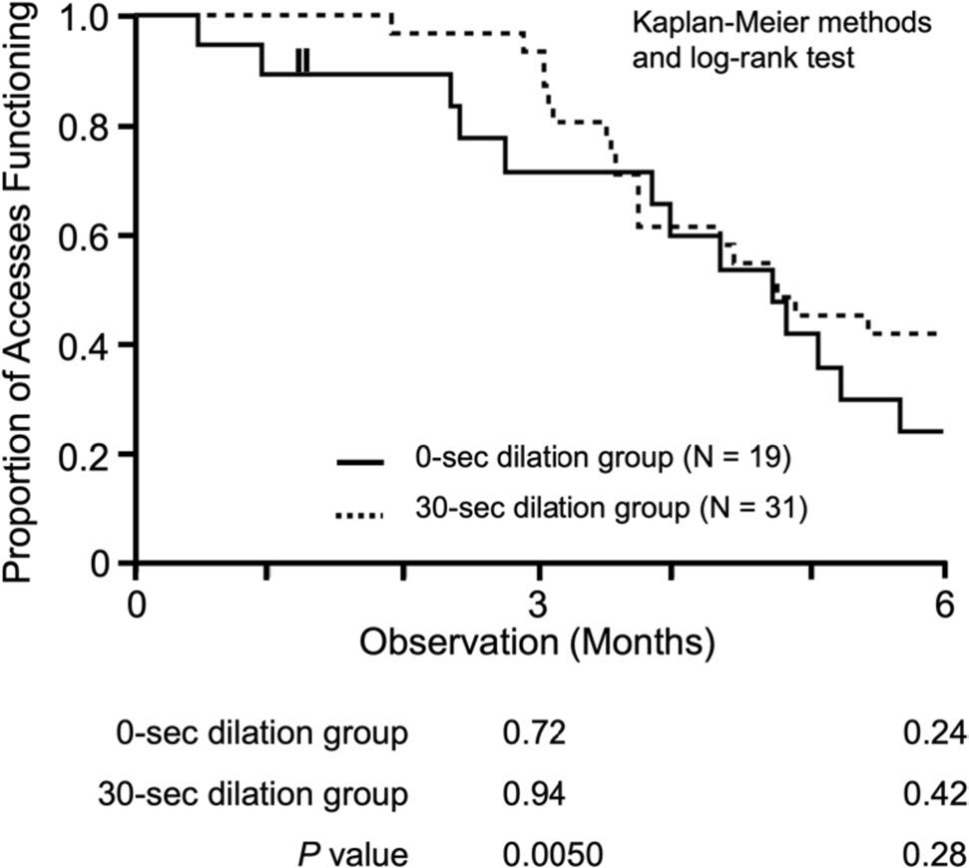
Comparison of 3- and 6-month patency rates between 0- and 30-s dilation groups. The comparison was performed using the Kaplan–Meier methods and the log-rank test. The solid line represents the 0-s dilation group, and the dotted line represents the 30-s dilation group. The shorter vertical bars indicate censoring. The analysis revealed a significant difference in the 3-month patency rate (*P* = 0.0050). However, there was no significant difference in the 6-month patency rate between the two groups (*P* = 0.28)

To clarify the effect of dilation time on the patency rates at 3- and 6-month, Kaplan–Meier methods and log-rank tests were used to conduct a comparative analysis. The 3-month patency rate showed a significant difference between the 0- and 30-s dilation groups (72% and 94%, respectively; *P* = 0.0050), whereas, at 6 months, there was no significant difference between the two groups (24% and 42% in the 0- and 30-s groups, respectively; *P* = 0.28) (Fig. 2).

### Clinical factors associated with the 3-month patency rate

Given that the 3-month patency rate is higher in the 30-s dilation group compared to the 0-s dilation group, we next investigated whether this beneficial effect was independent of other clinical factors. Multivariate Cox proportional hazard model revealed that 30-s dilation (hazard ratio (HR) 0.027; *P* = 0.0072), diameter of proximal site of stenosis (HR 0.32; *P* = 0.031), and dilation pressure (HR 0.63; *P* = 0.014) were independently associated with the 3-month patency rate; however, age (*P* = 0.15), presence of DM (*P* = 0.97), diameter (*P* = 0.081) and length (*P* = 0.77) of stenosis, and diameter of the distal site of stenosis (*P* = 0.35) showed no significant association (Table 2).

**Table 2:** Clinical factors associated with 3-month patency rate

	Hazard ratio [95% CI]	*P* value
Age (per 1-year increase)	0.93 [0.84–1.03]	0.15
Diabetes mellitus, presence	1.05 [0.08–13.92]	0.97
Treatment, 30-s group	0.027 [0.002–0.38]	0.0072
Stenosis diameter		
Site of stenosis (per 1-mm increase)	0.20 [0.02–0.95]	0.081
Proximal (per 1-mm increase)	0.32 [0.08–0.80]	0.031
Distal (per 1-mm increase)	1.25 [0.74–2.04]	0.35
Stenosis length (per 1-mm increase)	1.01 [0.95–1.07]	0.77
Pressure (per 1-atm increase)	0.63 [0.38–0.86]	0.014

Continuous data are summarized as the median [interquartile range] and categorical data as an absolute value (percentage)

*CI* confidence interval

### Comparison of dilation pressure between previous and the present PTA in both 0- and 30-s dilation groups

**Fig. 3:**
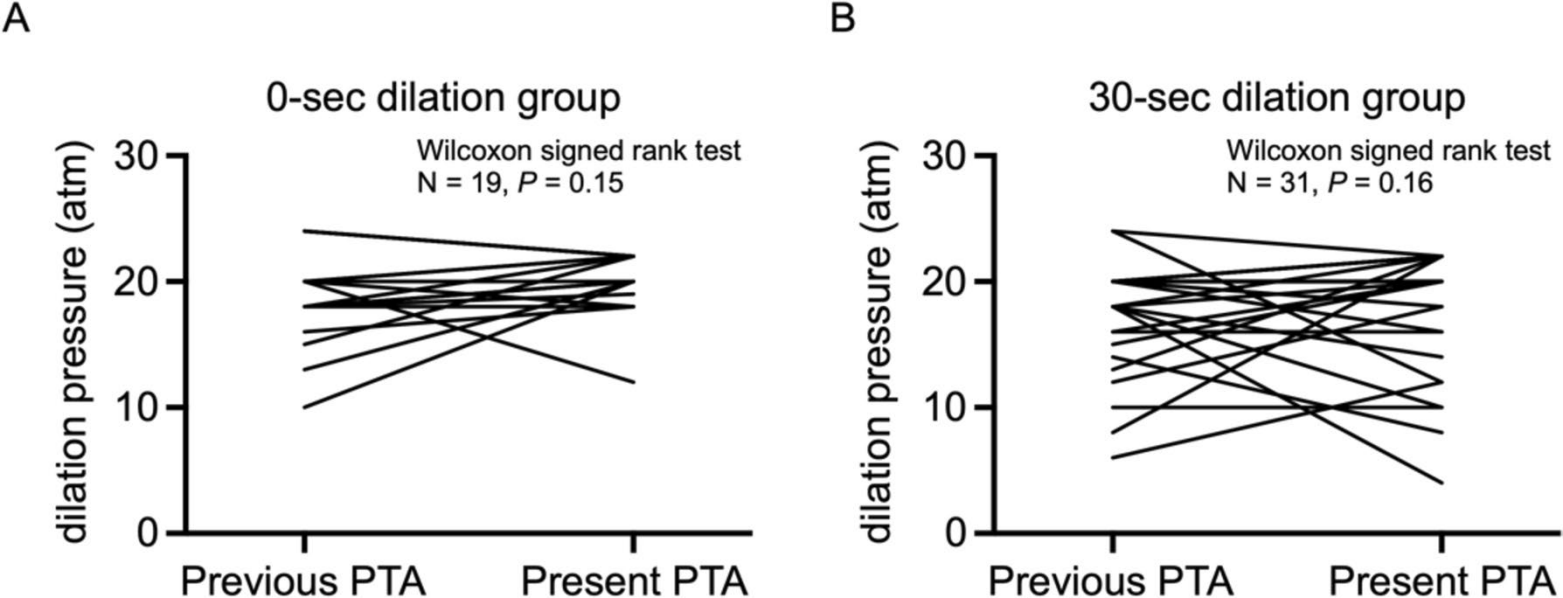
Comparison of dilation pressure between previous and present PTA in both 0- and 30-s dilation groups. The comparison of dilation pressure between 0-s (**A**) and 30-s (**B**) dilation groups was conducted using the Wilcoxon sighed-rank test. There was no significant difference in dilation pressure between previous and present groups within both the 0-s (**A**) and 30-s (**B**) dilation groups (*P* = 0.15, *P* = 0.16, respectively). *PTA* percutaneous transluminal angioplasty

To determine the difference in dilation pressure between the previous and present PTA procedures, we next compared the balloon pressures used in both 0- and 30-s dilation groups. The Wilcoxon signed-rank test revealed that there was no significant difference between the two groups (*P* = 0.15, *P* = 0.16, respectively) (Fig. 3A, B).

### Comparison of 3- and 6-month patency rates between previous and present PTA in both 0- and 30-s dilation groups

**Fig. 4:**
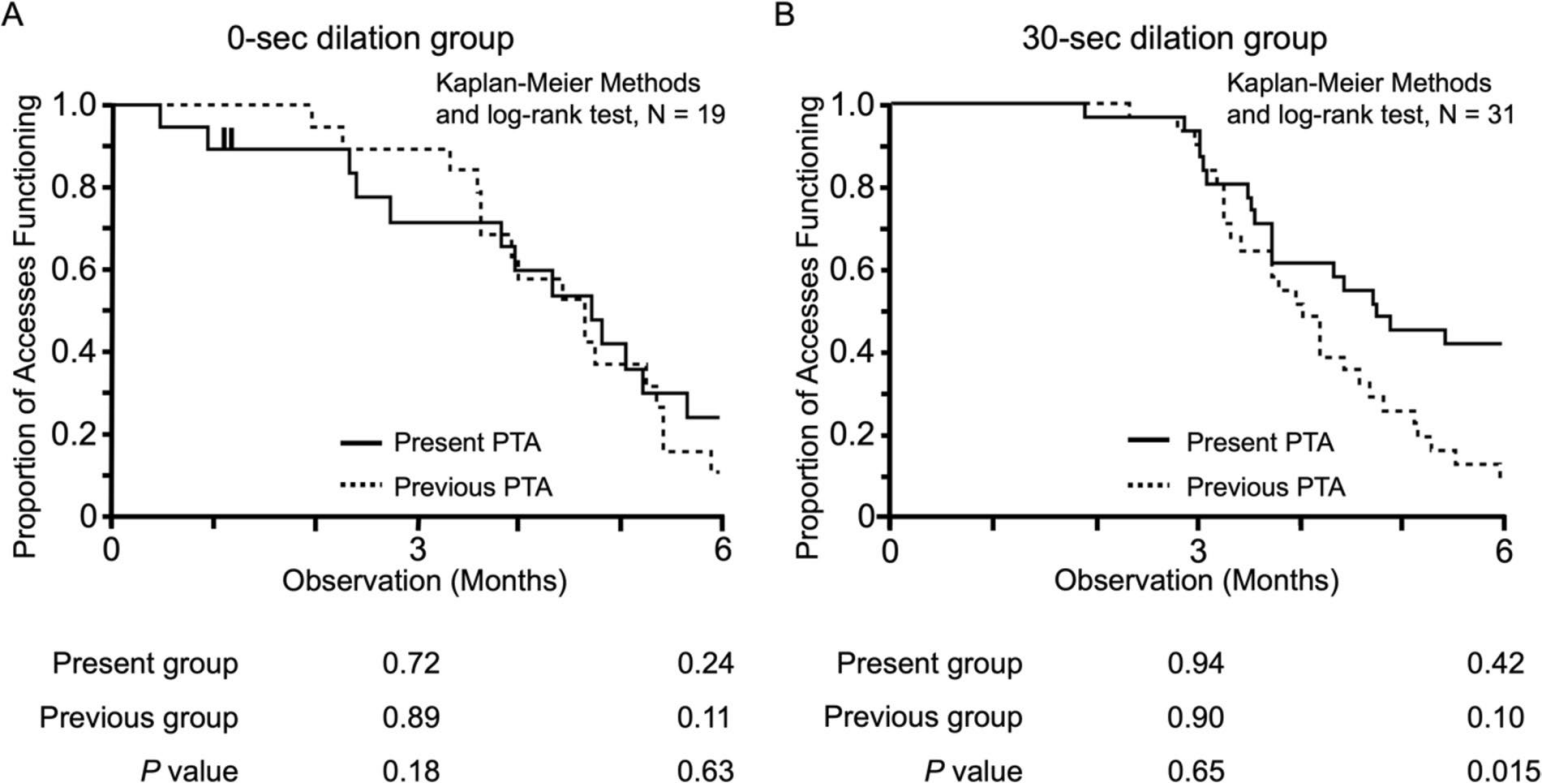
Comparison of 3- and 6-month patency rates between the previous and the present PTA in both the 0- and 30-s dilation groups. The comparison of 3- and 6-month patency rates between 0-s (**A**) and 30-s (**B**) dilation groups was performed using the Kaplan–Meier methods and the log-rank test. The solid line indicates the present PTA group, and the dotted line indicates the previous PTA group. The shorter vertical bars indicate censoring. **A** The 0-s dilation group appeared to exhibit a slightly lower 3-month patency rate without significant differences (*P* = 0.18). However, the 6-month patency rate did not show significant differences between the two groups (*P* = 0.63). **B** The 30-s dilation did not significantly improve 3-month patency rate (*P* = 0.65). On the other hand, the 6-month patency rate in present PTA was significantly better than that in the previous PTA (*P* = 0.015). *PTA* percutaneous transluminal angioplasty

In comparison of the patency rates between previous and present PTA at 3- and 6-months in both 0- and 30-s dilation groups, 0-s dilation appeared to exhibit a slightly lower 3-month patency rate without significant differences (*P* = 0.18), while 6-month patency rate did not show significant differences between the two groups (*P* = 0.63; Fig. 4A). On the other hand, 30-s dilation did not significantly improve 3-month patency rate within previous and present PTA. However, 6-month patency rate in present PTA (42%) was found to be significantly better than that in previous PTA (10%) in 30-s dilation groups (*P* = 0.015; Fig. 4B).

### Comparison of the visual analog scale (VAS) between 0- and 30-s groups

**Fig. 5:**
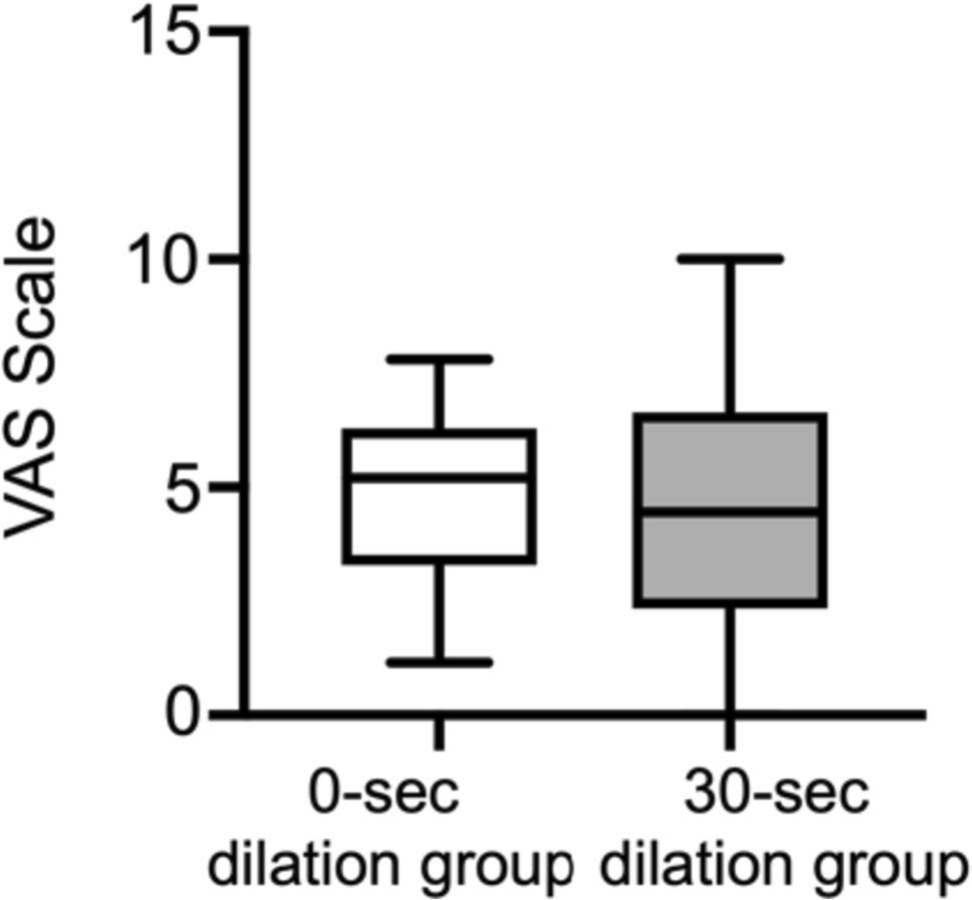
Comparison of Visual Analog Scale (VAS) between the 0- and 30-s dilation groups. The comparison of Visual Analog Scale (VAS) between 0- and 30-s dilation groups was carried out using the Mann–Whitney *U* test. There was no significant difference between the two groups (*P* = 0.51)

To evaluate the level of pain during PTA procedure, we compared the VAS scores between 0- and 30-s dilation groups. The Mann–Whitney *U* test revealed no significant difference in VAS scores between the two groups (*P* = 0.51) (Fig. 5).

## Discussion

We performed a prospective, multicenter, randomized, 2-group comparison study to identify the effect of dilation time on patency rates in HD patients with restenosis within 6 months. Of the 51 patients enrolled, 1 patient in the 30-s dilation group was excluded due to not undergoing prior PTA within 6 months. Finally, 50 patients were included in the analysis. During the observation period, 2 patients died from pneumonia and myocardial infarction before the end of the follow-up period. Herein, we have demonstrated that 3-month patency rate is higher in 30-s dilation group compared to 0-s dilation group, but the 6-month patency rate does not show a significant difference between 2 groups. In multivariate Cox proportional hazard model, 30-s dilation, diameter of proximal site of stenosis, and dilation pressure are independently associated with the 3-month patency rate. Dilation pressure between previous and present PTA shows no significant difference in both 0- and 30-s dilation groups. In comparison of the patency rates between previous and present PTA, 6-month patency rate of present PTA in 30-s dilation group is significantly better than that of previous PTA. VAS scores do not show a significant difference between the two groups. These findings suggest that 30-s dilation potentially has an advantage over 0-s dilation in the 3-month patency in HD patients with stenosis within 6 months after PTA.

We show that 3-month patency rate in 30-s dilation group is higher than that in 0-s dilation group, while 6-month patency rate does not show a significant difference between 2 groups. In this study, we enrolled the patients who underwent PTA for stenosed AVF within 6 months from the previous PTA, and therefore restenosis may occur within a short period. The presenting data suggest that the beneficial effect of 30-s dilation does not continue for the 6-month in HD patients with restenosis within 6 months. However, we show that 30-s dilation improves 3-month patency. As mentioned above, previous studies have demonstrated that patency rate does not differ between 1- and 3-min dilation [12] and that 30-s dilation exhibits better patency rate than 1-min dilation [13]. Taken together, these findings suggest that the 30-s dilation could be the best dilation method currently available.

The histology of venous stenosis is characterized as aggressive neointimal hyperplasia during wound healing [14–16]. Inadequate dilation during PTA procedures readily promotes venous stenosis, while excessive angioplasty [17, 18] also, in response to severe vascular injury, induces smooth muscle cell migration from the tunica media to the intima through mechanisms including oxidative stress, inflammation, and vascular endothelial dysfunction, resulting in the formation of neointimal hyperplasia and eventually venous restenosis [19–21]. The 30-s dilation reportedly shows better patency than the 1-min dilation [13], suggesting that severe neointimal hyperplasia due to excessive angioplasty may occur at balloon dilation times longer than 30 s. Furthermore, in this study, we show that the 30-s dilation exhibits a better 3-month patency rate than 0-s dilation, implying that the 0-s is likely to result in insufficient balloon dilation, and that the 30-s dilation may be well balanced between insufficient dilation and neointimal hyperplasia due to excessive dilation. Even more, in cases of limb injury, compression is a useful method to suppress tissue damage, along with rest, icing, and elevation. Similarly, 30-s dilation is considered as compression of injured vascular tissue after PTA, thereby improving the 3-month patency after PTA in patients with restenosis within 6 months. The presenting data suggest that the 30-s dilation could be a recommended strategy to improve the patency rate after PTA.

We show that 30-s dilation is independently associated with the 3-month patency, as well as diameter of the proximal site of stenosis, and dilation pressure, while age, diameter and length of stenosis, diameter of the distal site of stenosis, and presence of DM were not associated. Past studies have reported that advanced age, presence of DM, and severe stenosis are associated with poor patency rate after PTA [22, 23]. Additionally, we previously demonstrated that dilation pressure does not affect the 12-month patency rate through the comparison between 8- and 30-atm dilation [24]. That study shows that dilation pressure was associated with the 3-month patency rate. Therefore, we assume both dilation time and pressure may influence the patency rate after PTA more strongly than those other clinical factors. On the other hand, because balloon dilation causes severe pain, we assumed that patients in the 30-s dilation group exhibits higher VAS scores than those in the 0-s group. However, the presenting data show that VAS score does not differ between 2 groups. In this study, we performed local anesthesia before balloon dilation, suggesting that dilation time does not affect degree of pain if patients underwent local anesthesia.

Comparing the patency rate between previous and the present PTA, in 0-s dilation group, there are no significant differences in both the 3- and 6-month patency rates between the two groups. On the other hand, the 30-s dilation does not improve the 3-month patency rate, but it exhibits a better 6-month patency rate compared to the previous PTA. These findings suggest that the 30-s dilation exhibits a better patency rate. Regarding the dilation pressure, there are no significant differences between the previous and the present PTA in both the 0- and 30-s dilation groups. Additionally, complete dilation is not achieved in 26% of both the 0- and 30-s dilation groups (Table 1). We previously show that 24% of cases do not achieve complete dilation within 20 atm of dilation pressure, and that presence of residual stenosis does not affect the patency rates [24]. Collectively, we observed residual stenosis in 26% of patients, which does not seem to influence the resulting data that the 30-s dilation exhibits a better patency rate than the 0-s dilation.

The advantage of this study is its design as a randomized, multicenter prospective trial. We enrolled the patients with restenosis within 6 months from the previous PTA, and patency rate was evaluated in patients who underwent PTA at forearm of AVF. Thus, we examined the effect of dilation time in HD patients who exhibits similar condition. We use the SIRANUI EX or the OHICHO III, and their performance characteristics are similar. Therefore, effect of balloon catheters on patency rate does not need to take into account. However, a major limitation of this study is that it is characterized as a pilot study because of no previous study to compare patency rate between 0- and 30-s dilation groups, and therefore the actual effect of 30-s dilation should be confirmed through randomized control trial. Although we assigned the patients using the allocation table that included sex and presence of DM, more patients were assigned to the 30-s dilation group. Because we enrolled patients with restenosis at forearm of AVF, the beneficial effect of 30-s dilation may not apply to patients with AVG and those who underwent PTA for the first time. We lack information on inflation time during the last PTA. Moreover, we have a concern regarding the data representation, given that 51 cases out of 2029 total PTA cases may constitute a small sample size that might not be entirely representative of the general population of PTA cases. However, with regard to sample size, while the limited number of prospective studies on PTA, the enrollment of 51 cases is not a too small number referring to previous reports [12, 13].

In summary, we performed a randomized, multicenter prospective study to compare the patency rate between 0- and 30-s dilation in HD patients with restenosis within 6 months at forearm of AVF. We show that 30-s dilation exhibits better 3-month patency rate than 0-s dilation, while 6-month patency rate does not differ between 2 groups. Multivariate Cox proportional hazard analysis identifies that 30-s dilation, diameter of the proximal site of stenosis, and dilation pressure are independently associated with 3-month patency. Dilation pressure does not differ between previous and present PTA in 2 groups. Although 30-s dilation improves the 6-month patency rates, the 3-month patency rate does not differ in comparison with the previous PTA. Meanwhile, 0-s dilation appears to slightly worsen the 3-month patency rate without significant differences compared with previous PTA. VAS score does not differ between the 0- and 30-s dilation groups. Given that previous studies have demonstrated that 30-s dilation exhibits a higher patency rate, the presenting data suggest that 30-s dilation is recommended to potentially improve the patency rate of PTA.

## Declarations

### Approved number

Clinical Trial Protocol number: University Hospital Medical Information Network (UMIN) 000024331. Approval number of the ethics committee of Hiroshima University Hospital: C-113-1.
